# Enhanced Macrophage and Granulocytic Recruitment with Increased Neo-Angiogenesis in Chicken Embryo Yolk Sac Following In Ovo Probiotic Blend Administration

**DOI:** 10.3390/vetsci12090892

**Published:** 2025-09-15

**Authors:** Lucia Biagini, Stefano Pesaro, Livio Galosi, Donatella Volpatti, Danilo De Bellis, Alessandra Roncarati, Alessandra Gavazza, Giacomo Rossi

**Affiliations:** 1 School of Biosciences and Veterinary Medicine, University of Camerino, 62024 Matelica, Italy; lucia.biagini@unicam.it (L.B.); danilo.debellis@unicam.it (D.D.B.); alessandra.roncarati@unicam.it (A.R.); alessandra.gavazza@unicam.it (A.G.); giacomo.rossi@unicam.it (G.R.); 2 Department of Agricultural, Food, Environmental and Animal Sciences, University of Udine, 33100 Udine, Italy; stefano.pesaro@uniud.it

**Keywords:** yolk sac, in ovo inoculation, probiotics, chickens, macrophages

## Abstract

The yolk sac (YS) plays a pivotal role in avian embryonic development, contributing to both haematopoiesis and immune maturation. This study aimed to evaluate the effects of in ovo administration of a commercial probiotic blend (Slab51^®^) on YS cellular dynamics in chicken embryos. At embryonic day (ED) 18, Ross308 broiler eggs were injected with either the probiotic suspension (P) or sterile saline solution (C). YS tissues were sampled at 8, 12, 24 and 36 h post-inoculation for histological and immunohistochemical analysis. Probiotic-treated embryos exhibited a significant reduction in granulocytic foci within the YS, potentially reflecting enhanced peripheral migration of mature granulocytes. Concurrently, a progressive increase in Iba-1+ macrophages was observed in the probiotic group, suggesting accelerated macrophage differentiation. Immunophenotyping revealed a predominance of M1-iNOS+ macrophages across all timepoints, although a significant increase in M2-CD204+ macrophages was detected at 36 h in probiotic-treated embryos, indicating a possible anti-inflammatory shift. Furthermore, a marked increase in CD31+ endothelial cells in the probiotic group supports an associated rise in neo-angiogenesis. These findings suggest that in ovo probiotic administration modulates the YS microenvironment by promoting early macrophage recruitment, macrophage polarization and vascular remodeling. To our knowledge, this is the first report to demonstrate probiotic-induced structural and immunological alterations in the chicken embryo YS. These results provide novel insights into the early immunomodulatory effects of probiotics and highlight the potential of the YS as a key mediator of host–probiotic interaction during embryogenesis.

## Simple Summary

The application of the “in ovo” technique for administering substances to chicken embryo prior to hatching has been used over the last decade. The rationale behind the use of this technique for probiotic administration stems from its potential to provide early nutrition to the embryo, influencing gut development with positive microbial colonization and the development, structure and functionality of both innate and adaptive immune systems. A study was performed to evaluate the effects of the administration of a commercial probiotic blend in the amniotic fluid of chickens on embryonic day 18, with particular focus on the macrophagic population and phenotypic expression, granulocytic differentiation and neo-angiogenesis of the yolk sac.

## 1. Introduction

The application of the “in ovo” technique for administering substances, such as probiotic bacteria, to chicken embryo prior to hatching has been widely discussed over the last decade. Probiotics are defined as “live microorganisms which, when administered in adequate amounts, confer a health benefit on the host” [1]. The rationale behind the use of the in ovo technique for probiotic administration stems from its potential to provide early nutrition to the embryo, thereby influencing gut development and positive microbial colonization [2]. Chickens, in fact, are born with an incomplete development of the gut and an intestinal microbiota populated with a few bacterial families of maternal origin [3,4]. The administration of probiotic in the amniotic fluid enables direct contact with the chickens’ gastrointestinal and respiratory tract prior to hatching [5], contributing to the modulation of embryonic microbiota composition [6]. After hatching, a rapid increase in gut colonization is normally observed consequently to the contact with the environment, when both beneficial and pathogenic microorganisms become established in the intestinal tract of the animal, dramatically increasing the number of antigens presented to the immune system. More efficient digestion and absorption of nutrients and early probiotic exposure may lead to long-term productivity gains in rearing conditions. Concurrently, an immune response may be required to provide defense against certain ubiquitous pathogens (e.g., *Salmonella* spp., *E. coli*, *Eimeria* spp.). A substantial body of research has documented the impact of probiotics, specifically in ovo administration, on the development, structure and functionality of both the innate and adaptive immune systems [7,8,9]. The process of colonization of the immune organs and tissue is initiated during mid-embryogenesis; however, a pronounced maturation process occurs during the initial two weeks of life, particularly in the context of the primary lymphoid organs and Mucosal-Associated Lymphoid Tissue (MALT) [10]. In this phase, partial protection is offered by the antibodies of maternal origin, although it remains a period of particular vulnerability for the animal [11]. Consequently, the interest in the potential immune stimulation achieved by in ovo administration of various substances, primarily probiotics, has increased. Gut-delivered antigens are required for the immunological development of lymphoid organs, such as the Bursa of Fabricius, the spleen and the whole Gut-Associated Lymphoid Tissue (GALT) [12,13,14]. It is well established that components of the innate immune system, for instance macrophages and heterophils, have already colonized the gut at the time of hatching. Macrophages are fully active and able to perform their function through the production of cytokines and chemokines in the context of inflammation or antigen presentation, while heterophils are considered to be immature [7,15]. Macrophages are also directly involved in the dynamic interplay between the gut microbiome and the host, fundamental to facilitate the induction of tolerance, protection from pathogens and maintenance of gut homeostasis. Although the initial colonization of caecal tonsils by B and T lymphocytes occurs on ED 18, the substantial migration of these cells and subsequent proliferation in the MALT takes place during the initial week of life [12] and, as the number of B and T cells colonizing the organs increases, they also become functionally mature [16]. For this reason, the activity of macrophages and dendritic cells plays a pivotal role in the detection and presentation of both commensal and pathogenic microbial antigenic components and metabolites. Antigens that have been processed by macrophages are then exposed with the Major Histocompatibility Complex (MHC) II and presented to CD4+ T cells in the lamina propria or lymphoid follicles T cells [17,18]. These cells are induced to produce a variety of cytokines, including TGF-β and IL-10, which can drive T cells differentiation into various T cell subsets (Th1, Th2, Th17, or Treg). Among these, particularly involved in the introduction of the immune system to the component of the intestinal microbiota are Th2 and Treg which favor the dampening of inflammation while Th17 cells stimulate the differentiation of IgA-producing plasma cells and IgA secretion [19,20].

During embryogenesis the process of macrophage colonization in various tissues is initiated by the replication and differentiation of progenitors in the yolk sac (YS). The yolk sac is the oldest extra-embryonic membrane supporting embryogenesis [21,22]. It is predominantly recognized for its role in supporting the development of the embryo; in fact, there are several crucial functions that it performs. Primarily, it is involved in the process of erythropoiesis, as well as nutrient uptake and metabolism; furthermore, it facilitates the transfer of antibodies from the hen to the chicken [23]. In birds, YS tissue is the unique hematopoietic site from embryonic day (ED) 4 to ED 12. From this stage onwards, both the YS tissue and liver cooperate for erythropoietic function, thus being essential for primitive and definitive erythropoiesis [24,25]. In addition to the erythropoietic lineage, YS is one of the most significant sites for granulopoietic maturation. While its erythropoietic functions are partially replaced by bone marrow activity in proximity to hatching [26], granulopoietic differentiation remains apparently constant [24]. Furthermore, the vast majority of adult tissue-resident macrophages (e.g., Kupffer cells, microglia, Langerhans cells, alveolar macrophages and intestinal resident macrophages) derive from erythro-myeloid progenitor cells (EMPs) that originate from the YS [27]. These immune cells are the first line of defense in the event of early infections and, concomitantly, are involved in the stabilization of the gut microbiome. Thus, the correct colonization of the different tissues by resident macrophages can have long-lasting effects on the animal’s immune response. However, there remains a paucity of information regarding the potential impact of in ovo administration of probiotics on YS activity. Given its pivotal role in the erythropoiesis and maturation of both the granulocytic and macrophagic lineage, it can be hypothesized that beneficial effects on innate immunity observed after probiotic administration may also be related to the influence on the structure and functionality of the YS.

In this context, a study was performed to evaluate the effects of the administration of a commercial probiotic blend in the amniotic fluid of chickens on ED 18, with particular focus on the macrophagic population and phenotypic expression, granulocytic differentiation and neo-angiogenesis of the YS.

## 2. Materials and Methods

### 2.1. Probiotic Preparation and Treatments

The commercially available multi-strain probiotic Slab51^®^ (Ormendes SA, Jouxtens-Mezery, Switzerland) was used. It contains 200 billion lactic acid bacteria per 1.5 g of product, comprising the following strains: *Streptococcus thermophilus* DSM32245, *Bifidobacterium lactis* DSM 32246, *Bifidobacterium lactis* DSM 32247, *Lactobacillus acidophilus* DSM32241, *Lactobacillus helveticus* DSM32242, *Lactobacillus paracasei* DSM32243, *Lactobacillus plantarum* DSM32244 and *Lactobacillus brevis* DSM27961. The product was properly diluted using saline sterile solution to obtain a final concentration of 1 × 10^5^ CFU/100 μL of probiotics. The first group (P) received 1 × 10^5^ CFU of probiotic bacteria, diluted in 0.05 mL of saline sterile solution, while the remaining eggs, as control group (C), were inoculated exclusively with saline sterile solution.

### 2.2. Inoculation Procedure

Eggs of Ross308 broiler chickens were obtained from a commercial hatchery (Avizoo-Euroagricola s.s., Longiano, FC, Italy) and incubated under standard conditions (37.5 °C and 54% RH) in an incubator (mod. MG100/150 FIEM S.r.l., Como, Italy). Candling was performed at ED 8 to discharge dead embryos or unfertilized eggs. On ED 18, 80 fertilized and vital eggs were randomly allocated into two experimental groups (40 eggs/group) and subjected to the in ovo procedure as we previously described [28]. Briefly, after checking the exact position of the air chamber, all eggs were sanitized at the blunt end with 70% ethanol. A pilot hole was made with an 18-gauge needle to pierce only the shell without entering the air chamber. The needle was disinfected with 70% ethanol between injections. Administration into the amnion was performed with a 25-gauge needle (2.5 cm long) fitted to a 1 mL syringe, using a new sterile needle and syringe for each egg. After injection, the hole was sealed with glue, and all eggs were transferred to separate hatching baskets.

### 2.3. Sampling and Histological Examination

For each group, 8, 12, 24 and 36 h (T1, T2, T3, T4) after the in ovo procedure, the YS membrane was collected in 10 eggs. To avoid any suffering to the embryo, the eggs were placed for 30 min at −20 °C and then for 2 h at 4 °C. Next, all the embryos were sacrificed, and yolk membranes were fixed in 10% neutral-buffered formalin for 24 h. Tissue samples were routinely processed for histopathology [29]. Tissues were dehydrated in serial alcohol baths, cleared in xylene and embedded in paraffin wax, using an automatic tissue processor system. Histological sections, 3 μm thick, were obtained with a microtome and automatically stained with Hematoxylin and Eosin for the observation under the optical microscope (Leica DM2500, Wetzlar, Germany).

To assess granulocytic differentiation in the YS, ten microscopic fields were evaluated at 40× magnification in each section. The samples were evaluated not for the number of granulocytic cell precursors, but for the number of microscopic fields out of ten with foci of granulocytic differentiation (identified as groups of at least ten granulocytic cells) because of the uneven distribution of cells. This was performed to obtain a more representative evaluation of the samples. Therefore, all fields containing at least ten cells, whether single or grouped and corresponding to different stages of granulocytic differentiation, were counted. The mean values were then used to compare the presence of granulocytic differentiation in the two groups.

### 2.4. Immunohistochemical Staining

Paraffin-embedded tissue samples used for histological evaluation were cut to obtained 3 μm thick histological serial sections and placed on polarized slides. Immunohistochemistry was performed using avidine–biotine–peroxidase complex method (Vectastain Elite^®^ ABC Kit; Vector Laboratories, Inc., Newark, CA, US) with 3,3′-diaminobenzidine (Vector Laboratories, Inc., Newark, CA, US) as chromogen. Details of the primary and secondary antibodies and technical aspects are summarized in Table 1. Briefly, sections were subjected to specific pre-treatments for antigen retrieval. Non-specific binding sites were blocked by incubating sections at room temperature for 1 h with normal serum, obtained from the species corresponding to the secondary antibody, diluted in a solution of 1% bovine serum albumin, 1% polyvinylpyrrolidone and 1% tris-buffered saline (BSA-PVP-TBS). All sections were counterstained for 15 s with Harris’s hematoxylin. The specificity of the technique was demonstrated by replacing the primary antibody with PVP-BSA-TBS solution. The total number of macrophages in tissues was evaluated using an antibody against ionized calcium-binding protein molecule 1 (Iba1), a pan-macrophage marker. In addition, the polarization of macrophages into an M2 and M1 phenotype was identified using anti-macrophage scavenger receptor (MSR-A; CD204) and inducible nitric oxide synthase (iNOS) antibody, respectively. For vascular differentiation, the CD31 antibody, marker of mature endothelial cells, was used to determine the total number of micro-vessels. For all the samples, the total number of cells or micro-vessels expressing the marker was counted in five different microscopic fields at 40× magnification. Arithmetic means were calculated for each sample. The results obtained for iNOS+ and CD204+ cells were expressed as a percentage.

To validate the use of the anti-CD204 antibody in chicken samples, four controls were included: (i) omission of the primary antibody was performed to assess background staining from secondary reagent; (ii) an isotype control antibody (mouse IgG1 monoclonal, R312-MouseIgG1, ab280974, Abcam, Cambridge, UK) was used to evaluate non-specific binding related to antibody class; (iii) a non-sense antibody of the same species and isotype (mouse anti-Synaptophys in antibody, clone YE269, ab309493, Abcam, Cambridge, UK) was applied as an additional negative control; (iv) staining of mouse cutaneous tissue sections with the anti-CD204 antibody served as a positive control, as CD204 expression in this tissue is well established.

### 2.5. Statistical Analysis

Data were statistically analyzed using independent sample *t*-test to compare the treated and control group (95% CI). A *p* < 0.05 was considered significant. The D’Agostino Pearson test was employed to assess the normality of the data distribution. Statistical analysis was performed using a software package (MedCalc^®^Version 22.026©1993–2024, Ostend, Belgium).

## 3. Results

**Figure 1:**
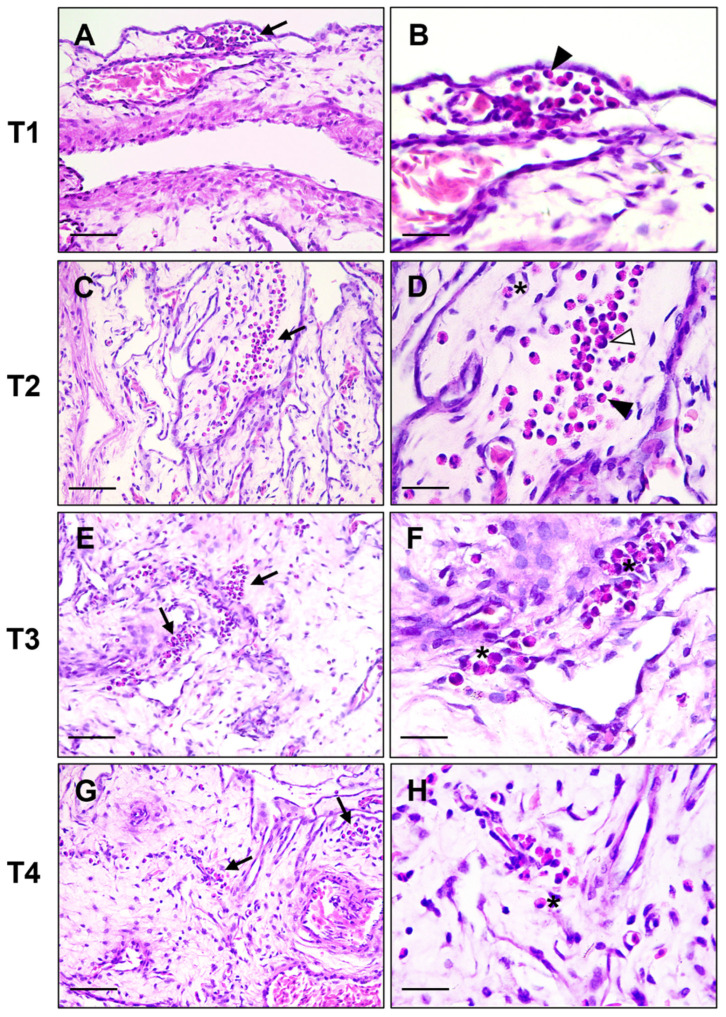
Chicken yolk sac. Representative images of granulocytic differentiation at four timepoints (T1–T4), shown without group distinction and presented at low and high magnification. Foci of granulocytic differentiation are indicated by black arrows. Different maturation stages are visible, including metamyelocytes (white arrowheads), band cells (arrowheads) and mature heterophils with eosinophilic granules (asterisks). Hematoxylin and Eosin stain. (**A**,**C**,**E**,**G**) Scale bar = 50 μm; (**B**,**D**,**F**,**H**) scale bar = 20 μm.

Histological evaluation of the YS of both experimental groups revealed the presence of granulopoietic foci in the mesodermal layer, with cells arranged in groups or interspersed in the tissue. Furthermore, is also evident a variable cell morphology, attributable to the various maturational stages of the granulopoietic line. Metamyelocytes and band cells represent the predominant cell types at T1 and T2, whereas there is a transition towards band cells and mature heterophiles with evident granulation at T3 and T4 (Figure 1). The evaluation of cell distribution in the YS tissue revealed a significantly higher presence of granulocytic foci in the control group (C) with a reduction in the final sampling (T4) for both groups (Table 2).

**Figure 2:**
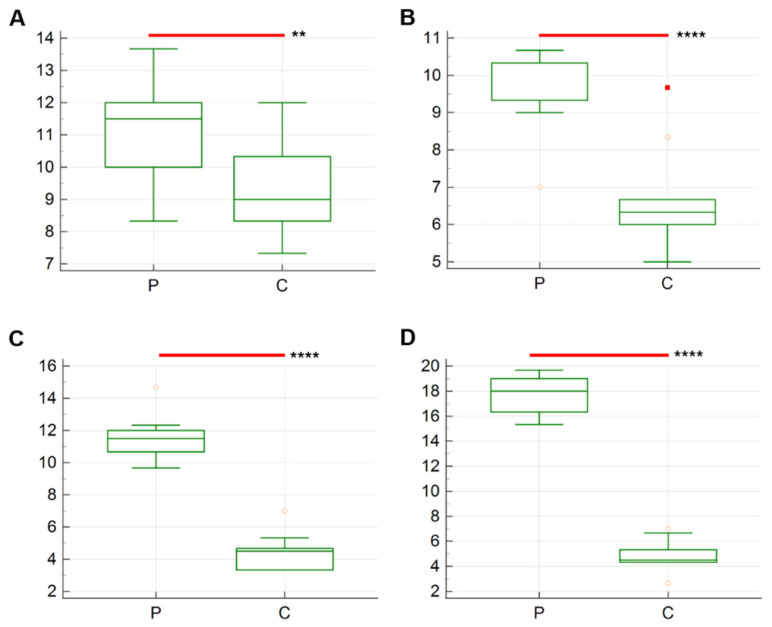
Boxplot showing the average number (*n* = 10) of anti-Iba-1 positive cells in the yolk sac tissues of probiotic-treated chickens (P) and control group chickens (C). (**A**) T1 (8 h post-inoculation); (**B**) T2 (12 h post-inoculation); (**C**) T3 (24 h post-inoculation); (**D**) T4 (36 h post-inoculation). The asterisks indicate the significant difference between the two groups. *p*-value, ****: <0.0001; **: <0.1.

**Figure 3:**
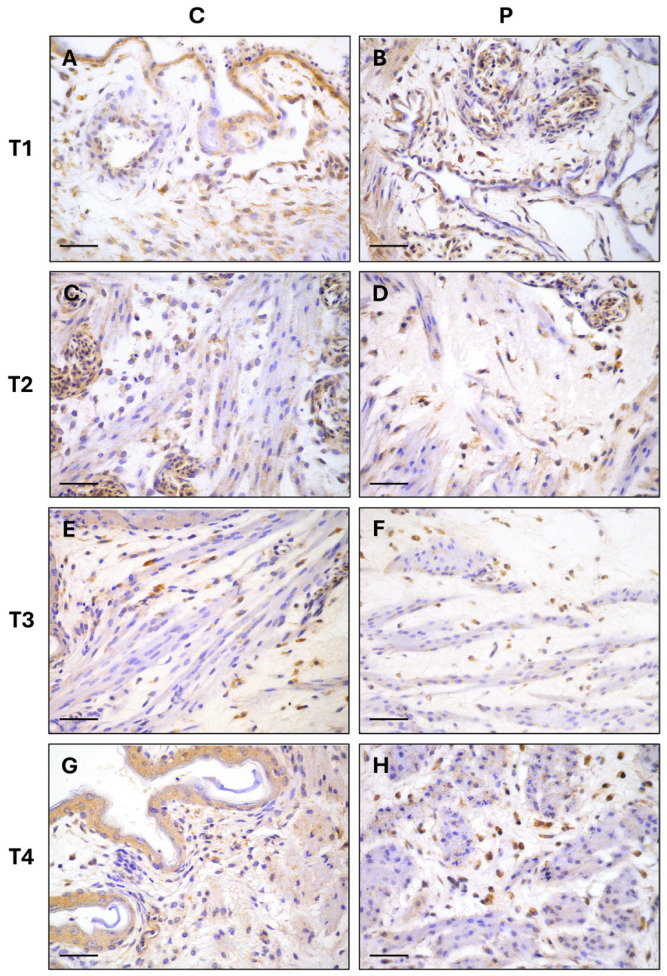
Yolk sac immunohistochemistry. Iba-1 expression in control group (C), (**A**,**C**,**E**,**G**) and in probiotic group (P), (**B**,**D**,**F**,**H**) in the different timepoints. Single or grouped cells displaying a granular to dense cytoplasmic immunolabeling are detected in the yolk sac tissue of both groups. The number of Iba-1+ cells is significantly higher at T4 in P group. IHC stain, Harris’s hematoxylin nuclear counterstain; scale bar = 50 µm.

The results of the average number of Iba-1 immunolabeled cells are described in Figure 2 and Figure 3. A statistically significant difference was identified between the two groups across the various sampling times. The average number of Iba-1+ cells in group P exhibited a progressive increase from T1 (9.8 ± 5.13) to T4 (23.57 ± 5.49), while the average results for group C remained constant at all timepoints.

**Figure 4:**
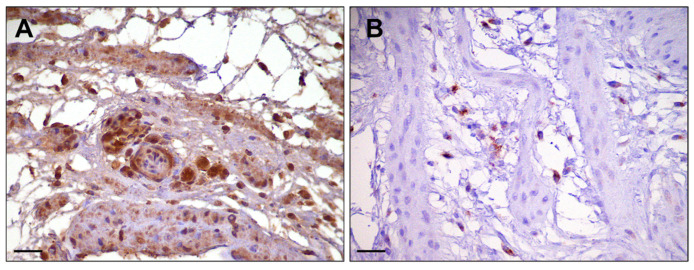
Immunohistochemical characterization of macrophage polarization in the yolk sac. Representative image of a sample from the probiotic group at T4: iNOS+ cells (M1 phenotype) (**A**); CD204+ cells (M2 phenotype) (**B**). IHC stain; Harris’ hematoxylin nuclear counterstain. Scale bar = 30 µm.

For the immunohistochemical characterization of macrophages polarization, iNOS was selected as a marker for macrophages with an M1 phenotype, while CD204 as a marker for macrophages with an M2 phenotype. The results for the percentage of positive cells are displayed in Table 3. Both markers were expressed by chickens YS macrophages, with a marked presence of iNOS positive cells in both groups at all timepoints (Figure 4). Single cells with a strong CD204 positivity were observed, predominantly at T3–T4, but still in a reduced percentage compared to M1. No significant differences were observed. M1 remained the predominant phenotype.

**Figure 5:**
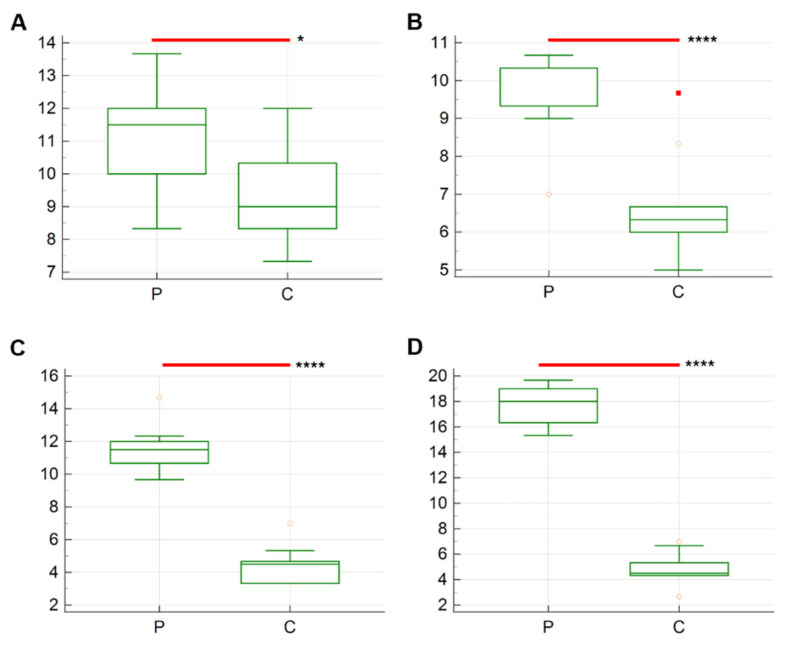
Boxplot showing the average number (n = 10) of anti- CD31+ cells in the yolk sac tissues of probiotic-treated chickens (group P) and control group chickens (group C). (**A**) T1 (8 h post-inoculation); (**B**) T2 (12 h post-inoculation); (**C**) T3 (24 h post-inoculation); (**D**) T4 (36 h post-inoculation). The asterisks indicate the significant difference between the two groups. *p*-value, ****: <0.0001; *: <0.05.

**Figure 6:**
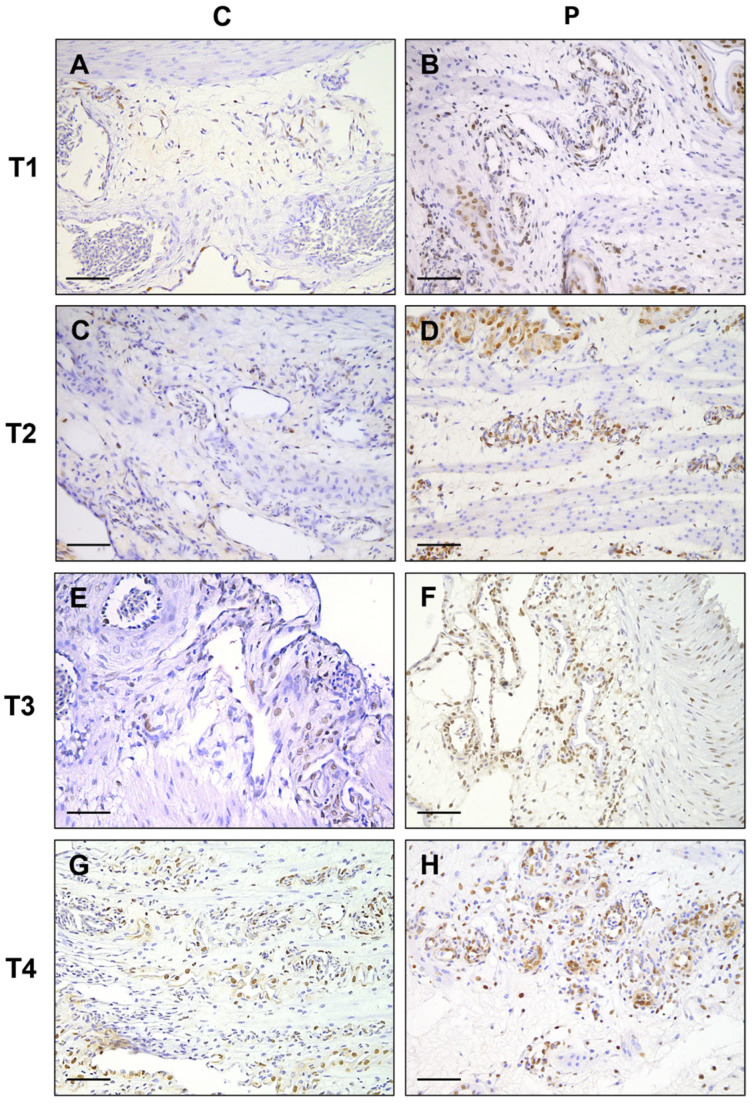
Yolk sac immunohistochemistry. CD31 expression in control group (C), (**A**,**C**,**E**,**G**), and in probiotic-treated group (P), (**B**,**D**,**F**,**H**). Grouped cells and small micro-vessels with a patent lumen show CD31+ immunolabeling, particularly evident at T4 in the yolk sac of P (H). IHC stain, Harris’s haematoxylin nuclear counterstain; scale bar = 50 µm.

The number of anti-CD31 positive cells in the different timepoints is presented in Figure 5. Significant differences are observed from T1 to T4, with higher number of cells stained positively in P group compared to C in all timepoints. The higher average results are registered for group P at T4 compared to C (17.80 ± 0.47 vs. 4.83 ± 0.39; *p* < 0.0001 (Figure 5D). In contrast, slight changes are observed in group C, whose results prove to be stable throughout the experiment (T1: 9.4 ± 0.51; T2: 6.6 ± 0.43; 4.46 ± 0.35; T4: 4.83 ± 0.39) (Figure 6).

## 4. Discussion

The use of in ovo administration as a means of providing nutrients to the embryo during the final stage of incubation has proven to be a subject of considerable interest [30]. This technique is arousing the interest of different stakeholders in the poultry supply chain, such as hatcheries managers who have to provide healthy and vigorous chicks, and poultry farmers who have to target high performance productions with the most favorable feed conversion rate.

Regarding probiotics, although a multitude of beneficial effects of this administration have been described, there are still numerous variables associated with the type of probiotic employed, dosage, site of administration and day of incubation selected [31]. Previous studies have evaluated the safety and efficacy of the commercial Slab51^®^ mixture as a feed supplement [32] or for in ovo administration in chickens [28]. The present study was conducted with the aim of furthering the understanding of the mechanisms underlying the interplay between the host immune system and probiotics, with a particular focus on YS.

The avian YS is a multifunctional extra-embryonic organ fundamental for embryonic development. Derived from the midgut of the developing embryo, it is composed of three cell layers: endoderm, mesoderm and ectoderm [23]. The endodermal layer consists of interconnected cells, the structure of which can be associated with intestinal tight junctions. This inner layer, in direct contact with the yolk, contains villous-like structures to increase its surface area and its high vascularization and non-specific phagocytosis are involved in nutrient absorption [33,34]. Although it is neither morphologically nor functionally part of the embryonic gut, its structure and organization suggest its primary absorptive function involves embryonic nutrition and metabolism, but also many aspects of immunity [33]. Maternal antibodies are concentrated in the yolk and transferred to the embryo during incubation, thereby providing it with passive immunity [35,36]. Additionally, it performs central erythropoietic and myelopoietic functions since, as reported by various sources, the development and maturation of a cell-mediated immune component such as macrophages or granulocytes takes place within [24,26].

The involvement of YS in granulopoietic differentiation has been a subject of debate, particularly regarding its contribution up to the conclusion of the incubation period. Previous studies have demonstrated that granulopoietic foci, comprising promyelocytes that subsequently mature into mature leukocytes, are present in abundance in the second half of chicken development [24]. At ED18, a co-existence of foci dedicated to erythropoiesis and granulopoiesis has been observed but separated into distinct niches [24]. While erythropoiesis undergoes a progressive decline, granulopoiesis remains constant in the YS, even after the onset of bone marrow activity [24,25]. The results of our study confirm these findings, observing a conserved presence of granulopoietic differentiation in the YS of both groups until the last sampling time, corresponding to ED 19.5. Previously, it has been observed that in ovo administration of different antigenic compounds can modulate the granulocytic response [37]. Moreover, the stimulation of the granulocytic and monocytic response using the cytokine Granulocyte–macrophage colony stimulating factor (GM-CSF) has been studied as an adjuvant to enhance humoral and T lymphocyte proliferation in order to increase the effect of vaccinations [38,39]. However, the impact of in ovo probiotic administration on YS granulopoietic proliferation and maturation remains to be clarified. The results obtained demonstrate a reduction in the number of granulocytic cells in the YS of probiotic-treated embryos. The difference between the two groups can be attributed to an earlier migration of mature granulocytes to other organs, attracted by the antigenic stimulation resulting from probiotics administration, a stimulus which is absent in the control group. Furthermore, the persistence of mature granulocytes within the YS highlights their involvement in the physiological reabsorption process that occurs at the end of the incubation phase.

Iba-1 is a marker frequently employed for the identification of histiocytic cells, thus serving as a reliable indicator of the number of macrophages present in the analyzed tissue [40]. The analysis of the YS collected at different timepoints showed a significantly higher presence of Iba-1+ macrophages in probiotic-treated embryos, compared to control samples. This finding was consistent across all timepoints, suggesting that early probiotic administration significantly promotes the maturation of YS myelopoietic stem cells, leading to the differentiation of these cells into mature macrophages. Our data also indicates that the average number of positive cells is notably higher in the probiotic group compared to the control at each sampling time. Conversely, the control group exhibited no variations in the YS tissue, with the mean number of Iba-1+ cells remaining largely constant. In ovo administration of probiotics stimulates the immune response, thereby increasing EMP activity in the YS, consequently leading to the colonization of tissue-resident macrophages in other organs. Perdiguero and colleagues [41] demonstrated that, in murine models, erythrocytes, granulocytes and monocytes are replaced by bone marrow-derived cells postnatally, whereas tissue-resident macrophages undergo no replacement during the first year of life. This observation indicates that the embryonic erythropoietic function of the YS plays a pivotal role in the development and subsequent maintenance of innate immunity. In line with this evidence, our hypothesis is that an early priming of these macrophages with a very broad and varied antigenic repertoire via Toll-like receptor (TLRs) may direct the pro-active or pro-tolerogenic activity of these central and multitasking cells of the immune system, even after birth, influencing the host immune response throughout life. Indeed, macrophagic immune components are already active before hatching at the time of in ovo administration [15]. Antigenic stimulation resultant to probiotic administration may increase cell migration from the productive reserves of the YS to the GALT, with particular attention on intestinal resident macrophages. This up-regulation of macrophagic maturation is particularly evident at T4, 36 h post administration. A previous manuscript already reported the stimulating effect or oral probiotic treatment on the number of intestinal macrophages [42], but the vast majority focus on their polarization, cytokine expression and phagocytic activity. Among their multiple features in fact, macrophages are well characterized for their extreme plasticity which is dependent upon environmental stimuli. Our findings demonstrate a high presence of iNOS+ cells in the YS tissue of both P and C groups. In the first attempt this inflammatory phenotype can be partially explicated by the technique itself, meaning that the inoculation into the amniotic fluid can stimulate an early inflammatory response in the YS tissue. Moreover, inflammation is a normal condition in the final stage of embryogenesis in the YS, which undergoes a physiological process of apoptosis and reabsorption. Only in probiotic-treated embryos there is an increase in CD204+ cells in the at 36 h post-treatment. Given the strict communication between the YS and intestinal tract, this reflect the same response activated by the gut immunity which is oriented to a dampening of the inflammation after the antigenic stimulation obtained with probiotics. The information available on the literature exhibits significant heterogeneity reporting the induction of M1 or M2 macrophages following the administration of diverse probiotic mixtures and strains [43], none of which describe the possible influence on YS reaction. The polarization into M1 or M2 phenotype can have both positive and negative effects on a specific organ depending on the conditions. For example, in murine models, cases of reduced severity of colonic macrophage infiltration after probiotic administration have not been associated with a dampening of pro-inflammatory M1 macrophages [44], indicating a multifactorial condition which drives this polarization. It has been suggested that the ability to drive a predominant M1 or M2 polarization can be associated with the bacterial strain used [45]. In any case, apart from phenotyping alone, the general cytokinetic profile expressed by both the polarized and non-polarized component in a tissue deserves closer examination [46].

Among the other functions, resident and infiltrating macrophages can also have a role in angiogenesis. Previous works attributed the angiogenetic activity to anti-inflammatory M2 but not pro-inflammatory M1 phenotype [47,48] although there are contrasting data showing macrophage-derived Vascular Endothelial Growth Factor (VEGF) production via both M1 and M2 origin [49]. The concomitant increase in the number of cells positive for both Iba-1 and CD31 suggests that not only the administered probiotics, but also the macrophages themselves, can stimulate the neo-angiogenesis in the tissue. CD31, a member of the immunoglobulin superfamily, is a 130 kDa transmembrane glycoprotein, also known as PECAM-1 (platelet–endothelial cell adhesion molecule) expressed on the surface of platelets, monocytes, macrophages and neutrophils, and is a constituent of the endothelial intercellular junction [50]. CD31 has been reported to participate in blood vessel formation during physiological and pathological processes, such as inflammation, wound healing, cardiovascular diseases and cancer [48]. The YS of group P showed higher isolated or grouped CD31+ cells at T1, while mainly small capillaries with a patent lumen were evident at T2-T4, which also correspond to increased CD204 percentage in probiotic-treated group. As already mentioned, the high degree of vascularization of the YS membrane is of fundamental importance both for its absorptive capacity and communication with other tissues. In this case, neo-angiogenesis is essential to support the increased cellular migration towards peripheral tissues, even if the actual increase in tissue colonization by resident macrophages after in ovo administration must be verified. It is certain that the interplay between macrophages and intestinal microbiota is pivotal in modulating the composition of gut microbiota and tolerance to its component, mainly through the recognition and presentation of microbial antigenic patterns [51,52]. Moreover, in birds, development and maturation of the Bursa Fabricii and GALT are strictly correlated to gut-delivered antigens [7,13,53]. Probiotics can stimulate the GALT immune system in chickens, primarily through the enhancement of TLRs signaling, the regulation of GALT responses, the promotion of dendritic cell-induced T cell hypo-responsiveness and the reinforcement of epithelial barrier integrity [54].

Our data confirm that the YS is important as the major intermediate erythropoietic and granulopoietic site where expansion and differentiation occur during chicken development. A marked induction of neoangiogenic activity and histiocytic production was observed after the administration of the probiotic-enriched inoculum, demonstrated by an expansion of erythrocytic and granulocytic lineages from 8 to 36 h post administration. This may facilitate the recognition and reaction to pathogenic microorganisms as part of the innate immune system’s first line of defense, while also modulating normal antigenic reactivity to commensal bacteria and promoting the creation of a tolerogenic environment and balanced microbiota. To our knowledge, this is the first study to highlight the morphological changes induced in the YS by in ovo administration of probiotics. Other studies are needed to acquire deeper information on the cytokine expression pattern established after probiotic stimulation both in the YS and in the other tissues such as the gut, as well as to study the reaction of the immune system to this early stimulation.

## Figures and Tables

**Table 1:** Antibodies used for immunolabeling of the yolk sac.

Primary Antibody	Host	Type	Secondary Antibody	Pre-Treatment	Dilution	Incubation (4°)	Code No.	Source
**Anti-Iba1**	Rabbit	P	Goat α Rabbit	Microwave, citrate buffer, pH6	1:300	Overnight	019-19741	FUJIFILM Wako Pure Chemical Corporation, Richmond, VA, USA
**Anti-CD204**	Mouse	M	Goat α Mouse	Microwave, citrate buffer, pH6	1:50	Overnight	KAL-KT022	TransGenic, Inc., Kobe, Japan
**Anti-iNOS**	Rabbit	P	Goat α Rabbit	Microwave, citrate buffer, pH6	1:300	Overnight	Ab3523	Abcam, Boston, MA, USA
**Anti-CD31**	Mouse	M	Goat α Mouse	Microwave, citrate buffer, pH6	1:100	Overnight	Ab119339	Abcam, Boston, MA, USA

M, monoclonal; P, polyclonal.

**Table 2:** Mean number of microscopic fields out of ten with presence of granulopoietic foci.

	P	C	Statistical Data
**T1**	5.5 ± 0.30	7.9 ± 0.43	*p* = 0.003
**T2**	5.1 ± 0.31	7.4 ± 0.26	*p* < 0.0001
**T3**	5.2 ± 0.35	7 ± 0.21	*p* = 0.0004
**T4**	3.5 ± 0.22	4.9 ± 0.23	*p* = 0.0004

Data are reported as mean ± standard error of the mean. P: treated group, inoculated with 100 μL of 1 × 10^5^ CFU/100 μL of probiotics; C: control group, inoculated with 100 μL of saline sterile solution; T1 (8 h post-inoculation); T2 (12 h post-inoculation); T3 (24 h post-inoculation); T4 (36 h post-inoculation). *p*: *p*-value.

**Table 3:** Average percentage of iNOS+ and CD204+ macrophages.

	P	C	Statistical Data
**iNOS**			
**T1**	96.8 ± 0.41	9.7 ± 0.51	*p* = 0.8820
**T2**	90.5 ± 0.89	90.6 ± 0.88	*p* = 0.9376
**T3**	86.7 ± 1.25	87.0 ± 1.37	*p* = 0.8738
**T4**	83.2 ± 1.30	86.4 ± 1.06	*p* = 0.0739
**CD204**			
**T1**	3.8 ± 0.51	4 ± 0.39	*p* = 0.7642
**T2**	9 ± 0.96	9.3 ± 0.91	*p* = 0.8241
**T3**	13.5. ± 1.36	13.8 ± 1.35	*p* = 0.8873
**T4**	13.9 ± 1.06	17.1 ± 1.37	*p* = 0.0821

Data are reported as mean ± standard error of the mean. **P**: treated group, inoculated with 100 μL of 1 × 10^5^ CFU/100 μL of probiotics; **C**: control group, inoculated with 100 μL of saline sterile solution; T1 (8 h post-inoculation); T2 (12 h post-inoculation); T3 (24 h post-inoculation); T4 (36 h post-inoculation). *p*: *p*-value.

## Data Availability

All data are included in the article.

## Abbreviations

The following abbreviations are used in this manuscript:

ED	Embryonic Day
YS	Yolk Sac
P	Probiotic group
C	Control group
MALT	Mucosal-Associated Lymphoid Tissue
GALT	Gut-Associated Lymphoid Tissue
MHC	Major Histocompatibility Complex
EMPs	Erythro-myeloid Progenitor Cells
CFU	Colony Forming Unit
BSA-PVP-TBS	1% bovine serum albumin, 1% polyvinylpyrrolidone, 1% tris-buffered saline
Iba1	Ionized calcium-binding protein molecule 1
iNOS	inducible nitric oxide synthase
